# Device-Driven Service Allocation in Mobile Edge Computing with Location Prediction

**DOI:** 10.3390/s25103025

**Published:** 2025-05-11

**Authors:** Qian Zeng, Xiaobo Li, Yixuan Chen, Minghao Yang, Xingbang Liu, Yuetian Liu, Shiwei Xiu

**Affiliations:** 1 State Key Laboratory of Petroleum Resources and Prospecting, China University of Petroleum (Beijing), Beijing 102249, China; zengqian@petrochina.com.cn (Q.Z.); lyt51@163.com (Y.L.); 2 Research Institute of Petroleum Exploration and Development, PetroChina, Beijing 100083, China; mgvmh22@petrochina.com.cn (M.Y.); xingbangliu@petrochina.com.cn (X.L.); xiusw_2024@petrochina.com.cn (S.X.); 3 School of Information Engineering, China University of Geosciences (Beijing), Beijing 100083, China; yixuanchen@email.cugb.edu.cn

**Keywords:** mobile edge computing, location prediction, service dynamic allocation, service deployment cost

## Abstract

With the rapid deployment of edge base stations and the widespread application of 5G technology, Mobile Edge Computing (MEC)has gradually transitioned from a theoretical concept to practical implementation, playing a key role in emerging human-machine interactions and innovative mobile applications. In the MEC environment, efficiently allocating services, effectively utilizing edge device resources, and ensuring timely service responses have become critical research topics. Existing studies often treat MEC service allocation as an offline strategy, where the real-time location of users is used as input, and static optimization is applied. However, this approach overlooks dynamic factors such as user mobility. To address this limitation, this paper constructs a model based on constraints, optimization objectives, and server connection methods, determines experimental parameters and evaluation metrics, and sets up an experimental framework. We propose an Edge Location Prediction Model (ELPM) suitable for the MEC scenario, which integrates Spatial-Temporal Graph Neural Networks and attention mechanisms. By leveraging attention parameters, ELPM acquires spatio-temporal adaptive weights, enabling accurate location predictions. We also design an improved service allocation strategy, MESDA, based on the Gray Wolf Optimization (GWO) algorithm. MESDA dynamically adjusts its exploration and exploitation components, and introduces a random factor to enhance the algorithm’s ability to determine the direction during later stages. To validate the effectiveness of the proposed methods, we conduct multiple controlled experiments focusing on both location prediction models and service allocation algorithms. The results show that, compared to the baseline methods, our approach achieves improvements of 2.56%, 5.29%, and 2.16% in terms of the average user connection to edge servers, average service deployment cost, and average service allocation execution time, respectively, demonstrating the superiority and feasibility of the proposed methods.

## 1. Introduction

With the development of technologies such as the Internet of Things, the amount of data, the number of devices, and traffic have increased dramatically. Due to the hardware limitations of mobile devices and the reliance on remote servers in traditional cloud computing, it is difficult to meet the requirements for high-performance and low-latency applications [1]. As a result, Mobile Edge Computing (MEC) has emerged, which can deploy resources at edge nodes to optimize performance [2]. Although existing research has made progress in service allocation [3], task offloading, and other aspects [4], many challenges still remain.

Most existing MEC methods rely on static models, which are primarily suited for idealized experimental settings and neglect the dynamic behavior of users and real-world application scenarios [5]. Specifically, in dynamic MEC environments, prediction algorithms suffer from poor accuracy and adaptability, as they fail to account for spatio-temporal dependencies, thereby increasing the uncertainty in service allocation and negatively impacting system performance and user experience [6]. Moreover, existing methods typically assume that edge users arrive synchronously, simplifying the user-service allocation problem into a constrained multi-objective optimization issue. However, these methods overlook the inherent irregularity and unpredictability of task request arrival times and geographical locations in real-world scenarios [7]. Without effective service allocation strategies, MEC systems may encounter performance bottlenecks and other related challenges.

To overcome the limitations of existing research, this paper first models resource allocation, clarifying constraints and objectives, and designs server connections and two model architectures. Then, an innovative framework consisting of two core modules, user location prediction and dynamic service allocation, is constructed to optimize edge computing resource scheduling and task processing, improving system performance and adaptability. The specific contributions can be summarized as follows:

1. This study constructs the ELPM model by combining Spatial-Temporal Graph Neural Networks (ST-GNN) with the attention mechanism [8]. Since user location changes in mobile edge computing scenarios exhibit both temporal and spatial characteristics, which traditional methods find difficult to capture, ST-GNN can compensate for these shortcomings. The attention mechanism adaptively weights historical trajectories, significantly improving prediction accuracy, providing reliable data for service allocation, and reducing service migration loss.
2. On this basis, for the dynamic service allocation problem, in order to maximize the connection ratio between edge users and edge servers while minimizing service deployment costs while ensuring user experience, this paper designs the Mobile Edge Service Dynamic Allocation (MESDA) strategy based on an improved Grey Wolf Optimizer (GWO) [9]. By introducing a random factor and adaptive mechanism to strengthen the direction judgment in the later iteration, combined with prediction results, tasks are allocated in advance, reducing migration times, improving resource utilization, and lowering costs.

We conduct multiple sets of experiments to validate the effectiveness of the models and strategies proposed in this paper. A dataset containing 100,000 user trajectory data points is used to test the models, covering user locations, service requirements, and server resource capacities. Compared with mainstream models and algorithms, we evaluate the performance advantages and potential for improvement. The experimental results show that the ST-GNN-based user location prediction model significantly improves prediction accuracy compared to traditional methods, thus improving service allocation efficiency and accuracy. In terms of service allocation, the MESDA algorithm also performs excellently in multiple metrics, such as connection rate, delay optimization, and service deployment cost. Specifically, the high accuracy of location prediction provides reliable input for dynamic service allocation, and the high efficiency of the service allocation strategy further optimizes overall system performance.

In summary, this paper presents a comprehensive solution for mobile edge computing service optimization by proposing a user location prediction model that combines ST-GNN with the attention mechanism and the dynamic service allocation strategy based on the MESDA algorithm. The combination of the two adapts to complex environments, improves the user experience, system performance, and resource efficiency, reduces costs and delays, and has important theoretical and practical value.

The remainder of this paper is organized as follows. We review related work in Section 2. The system model is introduced in Section 3. The location prediction model ELPM and service allocation algorithm MESDA are presented in Section 4. The performance of ELPM+MESDA is evaluated in Section 5. We conclude the paper in Section 6.

## 2. Related Work

Mobile edge computing has a multi-layer architecture similar to cloud computing, but it needs to address specific problems such as platform management, application deployment, and user access [10]. Its core component, the edge cloud server, faces many technical challenges in terms of design, deployment, and management. For formulating dynamic service allocation schemes required by users in the MEC environment, location prediction can predict the user’s position at the next moment in advance, providing strong support for early service allocation [11]. Based on the research results of location prediction, many scholars have also carried out in-depth research on user migration and service collaboration, service selection and optimization, service migration and offloading, and other aspects [12].

### 2.1. Location Prediction

Regarding the trajectory prediction in the MEC scenario, there are currently numerous prediction methods covering different aspects including individual interaction, behavior analysis, and physical mechanisms [13]. Nowadays, most edge devices can conveniently obtain users’ historical trajectories by means of the Global Positioning System. Based on these historical trajectory data, common prediction methods can be specifically classified into statistical methods and deep learning approaches.

The statistical methods take the Autoregressive Integrated Moving Average model (ARIMA) as an example. This model predicts the position at the next moment by linearly combining errors, historical data, and the current and historical values at certain moments. Chao Yan et al. [14] proposed a method that treats historical QoS values at different time slots as a temporal sequence of QoS matrices, applies truncated Singular Value Decomposition (SVD) to extract compressed matrices, and integrates these matrices with the classical ARIMA to extend its application for simultaneous prediction of multiple QoS values. The ARIMA assumes that the time-series data are stationary, and non-stationary time series can be transformed through difference operations, but its effect on non-linear and complex time-series data is not good, and it needs to be combined with the Seasonal ARIMA (SARIMA) or exogenous variables to improve the accuracy. The Gaussian Process Regression model (GPR) uses the covariance matrix to calculate the posterior distribution of predicted values and performs regression analysis on prior data. Haichao Liu et al. [15] employed Sparse Gaussian Process Regression (SGPR) to efficiently and accurately predict the future trajectories of surrounding vehicles. To enhance the robustness of the prediction model, the authors introduced translation and rotation transformation strategies, which effectively simplified the prediction problem. Additionally, they implemented an instant evaluation algorithm to assess prediction performance and maintained a streaming dataset for incremental learning, enabling the model to adapt to dynamic driving environments. The performance of the model may be sensitive to the choice of kernel functions and hyperparameters, which may require extensive tuning to achieve optimal results. Its computational efficiency is still constrained by the complexity of Gaussian process calculations, particularly in scenarios with large-scale datasets or high-dimensional input features.

In deep learning methods, the Multilayer Perceptron (MLP) uses historical data to predict users’ future intentions. Chen et al. [16] trained the backpropagation neural network with traffic information and used the simulation results for traffic prediction. The MLP model has strong fitting ability, but its ability to process sequential information of time-series data is limited. The prediction effect can be improved by introducing temporal features or combining with LSTM. The Recurrent Neural Network (RNN) can handle variable-length sequence data, but the traditional RNN has gradient problems and has difficulty in capturing long-term dependencies. The LSTM solves this problem by introducing a gating mechanism and can handle long sequence data. Zeng et al. [17] propose a sequence-to-sequence deep long short-term memory network (SS-DLSTM) for 4D trajectory prediction in terminal airspace, utilizing historical trajectory data and a regularization method to improve accuracy and robustness.

### 2.2. User Migration and Service Collaboration

When the server fails, it is crucial to achieve optimal user migration and ensure service synergy. Du et al. [18] defined EUM as an integer programming problem. They considered low latency, a high percentage of migrated users, and low service deployment costs, proposed a new solution method to handle constraints and find the optimal or approximate optimal solution, and also introduced a heuristic method to improve the solution efficiency. Wang et al. [19] re-modeled the collaboration and service latency problems in mobile edge computing using the Markov Decision Process framework, and proposed an offline dynamic programming algorithm and an online reinforcement learning algorithm. The dynamic programming algorithm decomposes the problem to avoid repeated calculations to improve efficiency, and the reinforcement learning algorithm enables the agent to learn and optimize strategies through interaction so as to adapt to the environment and solve the service latency problem [20].

### 2.3. Service Optimization and User Allocation

The mobile edge computing environment is constrained by various factors, such as computing resources, network transmission bandwidth, and device power. Cang et al. [21] proposed an optimization framework for joint user scheduling and computation resource allocation in asynchronous MEC networks, aiming to minimize energy consumption by optimizing task offloading strategies. Specifically, edge devices strategically offload tasks based on the energy they collect, while the MEC server processes these tasks asynchronously according to their arrival. To solve this mixed-integer nonlinear programming problem, the Benders decomposition method is employed, dividing the problem into the primal problem and the master problem. The optimal scheduling and resource allocation scheme is obtained through iterative solving of both problems.

For user allocation, different scholars have carried out a series of studies. Zou et al. [22] considered the situation where user requests can be decomposed and executed by multiple edge servers, proposed the Task Decomposable Edge User Allocation (TD-EUA) problem, and provided two methods, TD-EUA-O and TD-EUA-H, which are based on integer linear programming, significantly improving the quality of the user experience. Panda et al. [23] aimed at the dynamic allocation problem of user service requests and edge servers in mobile edge computing, introduced users’ personalized preferences for service quality, and then designed a user-centered optimal allocation strategy and a real-time mobility-aware algorithm. Peng et al. [24] regarded the edge user allocation problem as an online decision-making and evolvable process, proposed the MobMig mobility-aware migration method, and proved its superiority over traditional methods by conducting experiments using real-world MEC datasets. Liu et al. [25] modeled the User Allocation in Overlapping Areas (OAUA) problem in the mobile edge computing environment as a multi-objective optimization problem and proposed a convex-hull-based Pareto boundary search algorithm to reduce the algorithm complexity.

### 2.4. Service Migration and Offloading

Chen et al. [26] considered deploying mobile user services as container virtual machines, formulated container migration strategies, and proposed a multi-user server migration strategy (DRLMSM) based on the deep reinforcement learning algorithm (DRL). Huang et al. [27] proposed a deep reinforcement learning-based method for the multi-user service offloading and bandwidth allocation problems in mobile edge computing, jointly optimizing service offloading decisions and bandwidth allocation to minimize the overall offloading cost. These two studies focus on the dynamic migration and offloading strategies of services in the edge computing environment and provide new ideas and methods for ensuring the efficient operation of services and the rational utilization of resources.

### 2.5. Reservoir Dynamic Analysis Scenario

We focus on exploring the potential applications of MEC in specific scenarios, taking the case of reservoir dynamic analysis as an example. By integrating deep learning with geological information, real-time monitoring and prediction of reservoir dynamics can be achieved [28]. For instance, by combining historical production data with geological models, it is possible to dynamically forecast future demand changes in oil fields and optimize extraction scheduling. The introduction of MEC provides computational resource support for reservoir dynamic analysis, enabling more precise real-time assistance for resource scheduling, equipment management, and production decision making in oil field operations. Hussain et al. [29] proposes an edge computing-based resource allocation model for smart oilfields, aiming to address the challenges of efficiency and safety in offshore oil extraction. The study focuses on designing a robust task allocation mechanism by considering connectivity, computational capacity, and resource intensiveness, efficiently assigning tasks to appropriate edge or cloud resources to meet the real-time requirements of applications.

In reservoir monitoring systems, MEC-based edge computing resources can analyze sensor data in real time, predict production trends and equipment demand, and optimize resource allocation [30]. This dynamic decision-making system, based on historical data, can be integrated with reservoir production data to adjust service deployment and resource scheduling in real time, ensuring maximum production efficiency. Hussain et al. [31] propose a robust smart oilfield solution based on edge computing system alliances. By deploying nearby or mobile micro data centers to handle emergency tasks, it addresses the high latency and instability issues of satellite communication transmission, especially for disaster-related tasks that require real-time responses. By capturing the uncertainties in communication and computation within the joint environment and allocating emergency tasks, the proposed solution increases the likelihood of completing tasks on time. Furthermore, MEC technology, by predicting the movement behavior of users (such as oil field workers), can anticipate changes in equipment demand, provide early warnings for potential equipment failures or manpower scheduling issues during extraction, and, thereby, improve both production efficiency and safety [32].

In conclusion, most studies regard the service allocation problem in the MEC context as an offline decision-making process, mainly relying on users’ real-time location information as the model input but ignoring dynamic characteristics such as user mobility behavior. Many methods assume that edge users arrive simultaneously, simplifying the problem into a constrained multi-objective optimization problem and solving it with static optimization algorithms. However, in reality, the arrival time and location of task requests are random, and traditional allocation schemes may incur additional waiting time. Moreover, when services are dynamically deployed, existing methods only rely on users’ instantaneous location information. Even the strategies that partially consider the dynamics of the edge environment fail to fully utilize users’ historical movement trajectory data, and there are deficiencies when making service allocation decisions by combining user mobility information.

In contrast, this paper’s work will focus more on mining user mobility behavior characteristics, making full use of historical movement trajectory data, and constructing a service allocation model that is more in line with the actual dynamic environment. By introducing advanced prediction algorithms and dynamic decision-making mechanisms, it aims to achieve breakthroughs in the accuracy, timeliness, and resource utilization efficiency of service allocation, making up for the deficiencies of existing research and providing better solutions for service allocation in the edge computing environment.

## 3. System Model

This study focuses on the dynamic service allocation problem of edge users in the Mobile Edge Computing (MEC) scenario, involving resource quantification of the edge server system, user location prediction, and dynamic service allocation deployment, etc., which needs to be dealt with by using specific numerical values and formulas. We adopt a quantitative analysis method, first defining the problem and then clarifying the constraints and optimization objectives.

### 3.1. Problem Definition

In the Mobile Edge Computing (MEC) environment, the dynamic service allocation faces two core challenges [33]. On the one hand, user mobility and demands are highly dynamic. For example, during the morning rush hour, the distribution and demands of urban users are complex, increasing the difficulty of service allocation. On the other hand, the edge computing resources (including computing, storage, and service coverage) are limited. When users cannot access the edge servers, they need to switch to the remote cloud, which will cause latency and service quality degradation [34].

In the context of a smart city, MEC is employed to optimize public transportation systems, with a focus on predicting user locations and efficiently allocating services. Edge servers are deployed at transportation hubs or fixed facilities, interacting with base stations like traffic monitoring stations through mobile communication networks [35]. Users, in this case, are mobile phone users traveling through the city, generating location data that is periodically transmitted to edge servers via Wi-Fi or 5G networks [36]. The feasibility of location prediction in this scenario is driven by the continuous collection of real-time data from users’ smartphones, which can enhance service allocation. Access to location data is in compliance with privacy protection regulations, processing only anonymized data, and can be accessed only with user consent.

Therefore, the system has two optimization objectives: One is to maximize the proportion of users connected to the edge servers to provide low-latency and high-quality services; the other is to minimize the service deployment cost by reducing the latency and resource consumption of service migration, optimizing the user experience. Therefore, this study defines the following scenario: In a specific area, there are *m* edge servers $S=\{s_{1},s_{2},\ldots ,s_{m}\}$ and *n* edge users $U=\{u_{1},u_{2},\ldots ,u_{n}\}$, where each edge server has a specific amount of remaining resources of different types and also has a set of specific services required by users. Different services consume different amounts of resources, and the remaining resources of edge servers and the resources required by edge users are represented by a vector 〈CPU, storage, and bandwidth〉.

### 3.2. Explanation of Key Parameters

In order to accurately describe and solve the dynamic service allocation problem of edge users, a series of key parameters are introduced, which play a central role in constructing the system model, formulating constraints, and achieving optimization objectives. The detailed explanations are summarized in Table 1.

Specifically, if the service $sc_{p}$ required by edge user *u* is included in a finite set $SC=\{sc_{1},sc_{2},\ldots ,sc_{v}\}$ containing *v* services, then *u* will be allocated a specific amount of resources $r_{p}=\langle r_{p}^{q}\rangle$. This paper only considers the case where each edge user requires only one specific service.

If edge user *u* is within the service coverage radius of edge server $s_{i}$, it indicates that $s_{i}$ can provide services to *u*, that is,

(1) $$d_{u,i}\leq cov_{i},\;\forall u\in U\left(s_{i}\right),\;\forall s_{i}\in S$$

where $d_{u,i}$ represents the geographical distance between *u* and $s_{i}$, and $U(s_{i})$ represents the finite set of edge users within the service coverage of $s_{i}$. However, the sum of all resources allocated by the edge server to the edge users within its service coverage cannot exceed its remaining resources $a_{i}$, so we have

(2) $$\sum_{u\in \tilde{U}\left(s_{i}\right)}r_{sc_{u}}^{q}\leq a_{i}^{q},\;\forall s_{i}\in S,\;\forall q\in D,\;sc_{u}\in SC$$

In the actual service allocation decision making, one of the important goals of the system is to minimize the migration cost $mc_{u}$ of edge users, which consists of the following two parts: the service deployment cost $dc_{u}$ and the delay $h_{u}$ between the edge user and the connected edge server or remote cloud. It is specifically expressed as follows:

(3) $$mc_{u}=\left\{\begin{array}{cc} dc_{u}, & \mathrm{if}\;u\;\mathrm{is}\;\mathrm{connected}\;\mathrm{to}\;a\;\mathrm{feasible}\;\mathrm{edge}\;\mathrm{server}\\ h_{u}, & \mathrm{if}\;u\;\mathrm{is}\;\mathrm{connected}\;\mathrm{to}\;a\;\mathrm{remote}\;\mathrm{cloud} \end{array}\right.$$

This paper abstracts the edge servers in a specific area as a graph, where the nodes in the graph represent edge servers, and the edges represent the connections between two edge servers. Then, the distance between two nodes in the graph is the number of hops on their shortest path, where the number of hops between adjacent and connected edge servers is 1, and so on. Therefore, the delay between $s_{i}$ and $s_{j}$ can be represented by the distance between the corresponding two nodes in the graph, denoted as $h_{ij}$. And because $sc_{u}$ can be owned by one or more edge servers, in order to reduce $dc_{u}$, $s_{j}$ should be the edge server closest to *u* among $s_{i}$, where the calculation of $dc_{u}$ is as follows:

(4) $$dc_{u}=\left\{\begin{array}{cc} \min\{h_{ij}\}, & \mathrm{if}\;u\in \tilde{U}\left(s_{i}\right),\;s_{j}\in S\left(sc_{u}\right)\\ 0, & \mathrm{if}\;s_{i}\;\mathrm{and}\;s_{j}\;\mathrm{are}\;\mathrm{the}\;\mathrm{same}\;\mathrm{edge}\;\mathrm{server} \end{array}\right.$$

Here, $\tilde{U}\left(s_{i}\right)$ represents the finite set of edge users whose required services are successfully allocated to the edge server $s_{i}$, and $S(sc_{u})$ represents the finite set of edge servers that own the service $sc_{u}$ required by the edge user *u*. At the same time, in order to avoid some excessive delays in the service allocation process, $sc_{u}$ can only be allocated from nearby edge servers within a limited number of hops. The maximum value of the maximum hop count is denoted as $h_{max}$. In this paper, $h_{max}$ is set to 6, which means that $sc_{u}$ can only be allocated from edge servers within a distance of 0 to 5 hops, satisfying

(5) $$dc_{u}<h_{max},\;\forall u\in \tilde{U}\left(s_{i}\right)$$

The service dynamic allocation strategy in this paper is defined as a vector $M=\langle x_{u,i}\rangle$, where $x_{u,i}\left(u\in U,\;s_{i}\in S\right)$ indicates whether the edge user *u* is connected to the edge server $s_{i}$, and its value-taking rule is

(6) $$x_{u,i}=\left\{\begin{array}{cc} 1, & \mathrm{if}\;u\;\mathrm{is}\;\mathrm{connected}\;\mathrm{to}\;s_{i}\\ 0, & \mathrm{if}\;u\;\mathrm{is}\;\mathrm{not}\;\mathrm{connected}\;\mathrm{to}\;s_{i} \end{array}\right.$$

The domain of the variable set *M* is $I\left(x_{u,i}\right)=\left\{0,1\right\}$, which is applicable to ∀u∈[1,n], ∀i∈[1,m]. At the same time, an indicator $y_{i,p}\left(1\leq i\leq m,\;1\leq p\leq v\right)$ is introduced to indicate whether the edge server $s_{i}$ owns the service $sc_{p}$. Before performing trajectory prediction based on the user’s historical location, the value of each $y_{i,p}$ is pre-determined according to the service operator’s service dynamic allocation strategy:

(7) $$y_{i,p}=\left\{\begin{array}{cc} 1, & \mathrm{if}\;s_{i}\;\mathrm{owns}\;\mathrm{the}\;\mathrm{service}\;sc_{p}\\ 0, & \mathrm{if}\;s_{i}\;\mathrm{does}\;\mathrm{not}\;\mathrm{have}\;\mathrm{the}\;\mathrm{service}\;sc_{p} \end{array}\right.$$

### 3.3. Constraints and Optimization Objectives

In the mobile edge computing scenario, based on the previously defined problems and key parameters, in order to achieve efficient and reasonable dynamic allocation of edge user services, it is necessary to further refine the constraints and establish corresponding optimization objectives.

However, the computing resources of the edge server are limited. Too many users will cause the edge server to be overloaded. The specific constraint modeling in this study is as follows:

(8) $$x_{u,i}\cdot d_{u,i}\leq cov_{i},\;\forall u\in \left[1,n\right],\;\forall i\in \left[1,m\right]$$

(9) $$\sum_{u=1}^{n}x_{u,i}\cdot r_{sc_{u}}^{q}\leq a_{i}^{q},\;\forall i\in \left[1,m\right],\;\forall q\in \left[1,d\right]$$

(10) $$dc_{u}=x_{u,i}\cdot \left(1-y_{i,sc_{u}}\right)\cdot \min\left\{h_{i,j}\right\},\;\forall u\in \left[1,n\right],\;\forall i,j\in \left[1,m\right]$$

(11) $$0\leq dc_{u}\leq 5,\;\forall u\in \left[1,n\right]$$

(12) $$\sum_{i=1}^{m}x_{u,i}\leq 1,\;\forall u\in \left[1,n\right]$$

Here, constraint (8) represents the proximity constraint, which ensures that only edge users within the service coverage range of the edge server can potentially connect to the server, restricting the connection relationship between users and servers in geographical space and avoiding unreasonable connection attempts. Constraint (9) represents the resource capacity constraint, which stipulates that the sum of various resources consumed by the services required by edge users connected to the edge server shall not exceed the capacity of the edge server itself. Constraints (10) and (11) represent that when services need to be dynamically allocated, the services required by edge users will be allocated from the nearest edge server, and the service deployment cost is limited to between 0 and 5 hops. Constraint (12) indicates that an edge user can only be connected to one edge server or a remote cloud.

Therefore, from the perspective of edge users, in order to provide them with a better service experience, the first optimization goal is to maximize the percentage of users connected to the edge server, and the other optimization goal is to minimize the average service deployment cost of each edge user. The modeling of these two optimization goals is as follows:

(13) $$\max\left(\sum_{u=1}^{n}\sum_{i=1}^{m}x_{u,i}/n\right)$$

(14) $$\min\left(\sum_{u=1}^{n}mc_{u}/n\right)$$

The calculation of $mc_{u}$ is as follows:

(15) $$mc_{u}=h_{u}+\sum_{i=1}^{m}x_{u,i}\times \left(dc_{u}-h_{u}\right)$$

The delay between edge users and remote clouds is usually much greater than the delay between two edge servers, that is, $h_{u}\gg dc_{u}$. Therefore, when the edge user *u* is successfully connected to an edge server, $\sum_{i=1}^{m}x_{u,i}=1$ at this time, which means that it is the minimum value of $mc_{u}$. Therefore, the more edge users connected to the edge server, the smaller the value of the optimization goal (14). So, there is a positive mutual influence relationship between the optimization goal (13) and the optimization goal (14). Maximizing the proportion of users connected to edge servers inherently minimizes the average service deployment cost, as the latency costs of edge server connections are significantly lower than those of remote cloud connections; conversely, minimizing deployment costs incentivizes a higher utilization of edge servers, forming a mutual reinforcement—enhancing either objective directly advances the realization of the other.

## 4. Service Allocation Algorithm Based on Location Prediction: ELPM and MESDA

In response to the frequent mobility of users in the mobile edge computing scenario, this paper proposes a dynamic service allocation strategy based on location prediction. This strategy includes the ELPM location prediction model, which predicts the location of users at the next moment based on their historical movement trajectories, the calculation method of service deployment cost, and the MESDA dynamic service allocation strategy carried out based on the predicted location.

### 4.1. ELPM Location Prediction Model

Considering the high mobility of users in mobile edge computing, accurate location prediction is crucial for optimizing service allocation. Traditional prediction methods (such as linear extrapolation or fixed-rule models) struggle to cope with the randomness and complexity of users’ movement paths. Although LSTM and RNN models in deep learning have advantages in time-series processing, they have deficiencies when integrating the spatio-temporal information of mobile edge computing scenarios. The early Spatial-Temporal Neural Network (ST-GNN) integrated spatio-temporal information within a single framework and processed three-dimensional data in the sample format of (b, s, t, v). However, essentially, the modeling of its temporal and spatial characteristics is relatively independent, and it cannot meet the prediction accuracy requirements in complex mobility scenarios.

In view of this, this study introduces the Spatial-Temporal Graph Neural Network (STGNN) to construct the ELPM model. In the mobile edge computing scenario, user check-in points are abstracted as nodes of the graph, and the traffic flow situation on the roads is defined as the directed edges of the graph. The out direction represents the outflow of the check-in point, and vice versa for the inflow. This graph structure can accurately reflect spatial features and the dynamic changes of user movement. Specifically, the time dimension is reflected through the dynamic changes of the edges, and the space dimension is described by the inflow and outflow relationships of the check-in points. Thus, it solves the problem of independent spatio-temporal information modeling in traditional models.

In the specific implementation process of the model, the graph convolutional neural network (GCN) and the recurrent neural network (RNN) are used to implement the functions of STGNN. At a certain moment *T*, the scene is represented as a graph G=(V,E,T), where *V* and *E* represent the nodes and edges in the graph, respectively. For the adjacency matrix *A* of the graph, the node feature matrix $H^{l}$ of the *l*-th layer, and the initial node feature matrix $H^{\left(0\right)}$, the hierarchical update of graph convolution follows the following formula:

(16) $$H^{\left(l+1\right)}=\sigma \left({\tilde{D}}^{-\frac{1}{2}}\tilde{A}{\tilde{D}}^{-\frac{1}{2}}H^{\left(l\right)}W^{\left(l\right)}\right)$$

where $H^{\left(l+1\right)}$ is the stage feature matrix of the l+1-th layer, σ is a nonlinear activation function, $\tilde{A}=A+I_{n}$ represents the adjacency matrix *A* plus the identity matrix *l*, and $\tilde{D}$ is a diagonal matrix whose diagonal element ${\tilde{D}}_{ii}$ is equal to the sum of the *i*-th row elements of $\tilde{A}$ and is used for normalization.

Based on the above formula, first, use GCN to update the spatial features in each node. At each time step *t*, the spatial features are calculated by the formula $S_{t}=GCN\left(H^{0},A\right)$. Next, use RNN to process the time-series features, and the formula is $H_{t}=RNN\left(S_{t},H_{t}-1\right)$. Finally, the prediction result is output through the linear layer $Y_{t}=W_{0}H_{t}+b_{0}$, where $W_{0}$ and $b_{0}$ represent the weight matrix and bias matrix of the linear layer, respectively.

Nevertheless, the model constructed through the above steps still has a fitting deviation problem in actual training. From the perspective of modeling, the combination of GCN and RNN can indeed integrate the spatio-temporal information of the data and perform modeling, but the gradient in the training process is composed of the superposition of two parts of losses, which makes the model lack an effective adaptive mechanism when processing time and space information, resulting in possible over-emphasis on learning information in a certain dimension during the training process. To effectively solve this problem, we introduce the attention mechanism to optimize the model. For each node *v* in the space, the node features are updated using the following formula:

(17) $$h_{v}^{\prime }=\sigma \left(\sum_{u\in N(v)\cup \{v\}}\alpha_{vu}W_{s}h_{u}\right)$$

where $h_{v}^{\prime }$ represents the updated feature vector of node *v*, N(v) represents the set of neighbor nodes of node *v*, and α is the attention coefficient, reflecting the attention weight of node *v* to neighbor node *u*. The above formula models the feature information in the spatial dimension, while for the temporal attention, the following formula is used to update the node features:

(18) $$h_{v,t}^{\prime }=\sum_{\tau =1}^{T}\beta_{t\tau }W_{t}h_{v,\tau }$$

where $h_{v,t}^{\prime }$ represents the updated feature of node *v* at time *t*, and β represents the attention weight of time *t* to time step τ.

In the model training process, the set of trainable parameters mainly includes the spatial dimension adjacency matrix $W_{s}$ and the temporal dimension adjacency matrix $W_{t}$, as well as the attention parameters α and β. This paper uses θ to represent the set of model parameters, that is,

(19) $$\theta =\{W_{s},W_{t},\alpha ,\beta \}$$

The update of model parameters is achieved through the following formula, where L is the loss function and η is the learning rate:

(20) $$\theta =\theta -\eta \times \nabla_{\theta }L$$

**Figure 1:**
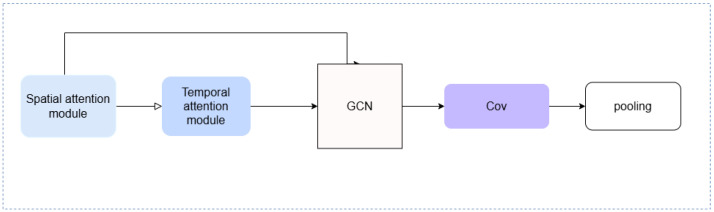
Spatio-temporal module structure.

In the process of specific implementation, this paper constructs a feature-mining module by building a Spatial-Temporal Neural Network block with the ability to mine spatio-temporal features. According to the derivation of the above formula, the construction of the feature mining module part is shown in Figure 1.

As you can see in the Figure 1, this module connects a time attention processing module and a space attention processing module in series as a feature converter for node feature update, and adjusts the bias of the time dimension and the space dimension in the data inference process through dynamic parameter connection. Then, the adjusted node features with spatio-temporal dimension bias are input into the GCN to model the structure of the graph. The edges and nodes of the graph are structured into embedding codes and connected through the adjacency matrix. The final output is the subgraph construction result in the modeling process.

**Figure 2:**
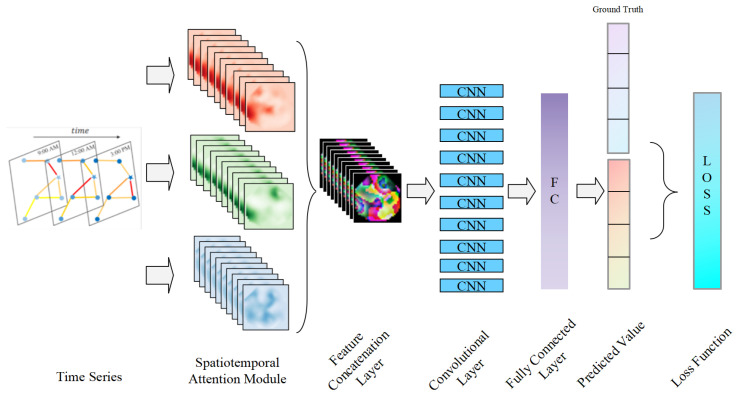
The specific implementation of the ELPM position prediction model.

In addition, we introduce OU noise into the model and split the input graph data into multiple inputs, which can not only enhance the robustness of the final modeling output result $Y^{\prime }$ of the model, but also enhance the degree of feature detail mining in the GCN stage. Next, the graph data are fused and modeled by paralleling multiple spatio-temporal attention module structures. The data are first updated through the spatial attention extraction module to update $h_{v}^{\prime }$ of each node, and then through the temporal attention module to incorporate temporal dimension information into the feature nodes. After these two steps, the attention parameters α and β are successfully obtained. For α and β in different OU processes, this paper also performs the splicing of embedding codes. The purpose of this step is to merge the features with different noises introduced and use the subsequent CNN module for autonomous feature mining. Finally, the FC layer is used to output the prediction result $Y^{\prime }$ and compared with the true value *Y* to calculate the prediction error. This process is shown in Figure 2 below.

In terms of model complexity, compared with the traditional attention-based spatio-temporal graph network (with a complexity of O(nlogn)), the method proposed in this study has a complexity of only O(1) when introducing OU process variables as noise. When performing vector merging, a binary merging method is adopted, with a complexity of O(logn). When performing the fully connected operation through the FC layer, although it involves the operations of the initialized parameter matrix and the CNN output matrix, the maximum complexity is only O(nlogn). Therefore, this method effectively controls the model complexity while ensuring the model performance, making it more feasible and efficient in practical applications.

### 4.2. The Calculation Method of Service Deployment Cost

This subsection mainly aims to minimize the average service deployment cost for each edge user and presents a specific quantification method for service deployment cost. This study ingeniously abstracts the edge server system as an undirected weighted graph, based on which, the service deployment cost is calculated. In this graph model, each edge server is regarded as a node, and the connection lines between servers are defined as weighted edges, with the weight uniformly set to 1. This modeling method simply and effectively reflects the connection relationship between edge servers and the basic cost structure of data transmission. The calculation of service deployment cost can be carried out in the following two parts:

1. First, use the graph theory depth-first search algorithm to model the relationship network among edge servers;
2. Then, use the Dijkstra algorithm to calculate the shortest path in the service allocation process (i.e., service deployment cost).
  First, abstract the relationship network composed of edge servers and define a *k*-cluster undirected relationship network G={V,E}, where *V* represents the global vertex set and *E* represents the initial edge set. The *i*-th cluster is represented as $G_{i}=\left\{V_{i},E_{i}\right\}$, where i=(1,2,⋯,k). Abstract the *i*-th cluster as a high-level node $V_{i}$. Whenever the number of connecting edges between two clusters is greater than zero, an abstract edge with a weight of 1 is established between them. All high-level nodes $V^{\prime }=\left\{V_{1},V_{2},\cdots ,V_{k}\right\}$ and the abstracted edges $E^{\prime }=\left\{v_{1},v_{2},\cdots ,v_{k}\right\}$ form the high-level relationship network $N=\{V^{\prime },E^{\prime }\}$ composed of edge servers.

The Depth-First Search (DFS) algorithm starts from a vertex in the graph, goes along a path until it cannot move forward, then backtracks to the previous node and continues to explore other paths until all nodes have been traversed. When constructing the high-level relationship network *N* composed of edge servers, elements in $V^{\prime }$ are grouped in pairs to obtain all possible combinations $C=\{\left(v_{1},v_{2}\right),\left(v_{1},v_{3}\right),\left(v_{1},v_{4}\right),\cdots ,\left(v_{k-1},v_{k}\right)\}$. The two high-level nodes of each element in *C* are respectively taken as the start point Start and the end point End. For example, for the element $\left(v_{1},v_{2}\right)$, $v_{1}$ is its start point and $v_{2}$ is its end point. In the high-level relationship network *N* composed of edge servers, the high-level nodes (clusters) that may be passed through between the start point and the end point are found according to the depth-first search. The pseudo-code of this part is shown in Algorithm 1. The elements in *C* are input into Algorithm 1 one by one, and finally, the data output by each is stored to obtain the result.

The Depth-First Search (DFS) algorithm needs to traverse all nodes and edges in the worst case, and its time complexity is O(V+E), where *V* represents the number of vertices and *E* represents the number of edges. *E* represents the number of edges. Since this algorithm uses a stack data structure to store the vertices to be visited and their neighboring vertices, in the worst case, such as in a chain graph structure, the number of elements to be stored can reach the level of O(V), so the space complexity of this algorithm is O(V).
**Algorithm 1** Finding all high-level nodes between start and end nodes in a graph.**Input:** A graph *G* with high-level nodes, start node Start, and end node End **Output:** Set of all high-level nodes between Start and End, shown in Path[]
  1:Initialize a queue *Q* and add Start to *Q*  2:Initialize an empty array Path[]  3:Initialize an empty set Visited  4:**while** queue is not empty **do**  5: Node ← Dequeue(Q)  6: Mark Node as visited (add to Visited)  7: **if** Node is End **then**  8:  Add Node to Path[]  9:  break10: **end if**11: unvisited_adjacent_nodes = Get unvisited adjacent nodes of Node (not in Visited)12: **if** unvisited_adjacent_nodes is not empty **then**13:  Randomly select one unvisited adjacent node and enqueue it to Q14: **else**15:  Remove Node from Q16: **end if**17:**end while**18:Output Path[]


After constructing the relationship network, the Dijkstra algorithm [37] is used to calculate the shortest path during the service allocation process, that is, the service deployment cost. The Dijkstra algorithm starts from a given starting vertex and adopts a greedy strategy, selecting the vertex that is closest to the starting vertex and has not been visited as the next vertex to visit each time, and continuously extends the path until reaching the target vertex. In the scenario of this study, any edge server is set as the starting node *s*, and an array dist is used to store the distances from all nodes in the graph to *s*. The value of dist[s] is initially 0, and for other nodes *v* in the graph, dist[v] is initialized to *∞*. During the execution of the algorithm, dist[v] will be continuously updated to store the shortest path distance between *s* and *v*. The pseudo-code of this algorithm is shown in Algorithm 2.

The time complexity of this algorithm is O(ElogE), because the minimum heap it uses can reach the O(E) level at most, and each element needs to be taken out from it once; its space complexity is O(N+E), where O(N) is the space used to store dist, and O(E) is the space used to store the adjacency list of the graph and the minimum heap.
**Algorithm 2** Calculate service deployment cost using Dijkstra’s algorithm.**Input:** Dijkstra(G, dist[], s) where G is the graph, dist[] stores distances from all nodes to s,   and s is the starting node **Output:** The shortest path p
  1:Initialize dist[s] to 0, and dist[v] to infinity for all other nodes v  2:**for** n times **do**  3: u ← the unvisited node with the smallest dist[u]  4: Mark u as visited  5: **for** all nodes v that can be reached from u **do**  6:  **if** v is not visited and dist[v] > dist[u] + cost(u, v) **then**  7:   dist[v] ← dist[u] + cost(u, v)  8:  **end if**  9: **end for**10:**end for**11:Output the shortest path p from the start node s to node v


Algorithms 1 and 2 are crucial components of the overall framework, ensuring the system operates efficiently. Algorithm 1 uses BFS to identify all high-level nodes between the start and end points, providing essential path information for service allocation and location prediction. It works in conjunction with the location prediction module, helping to make service allocation decisions by determining the potential user locations. Algorithm 2, on the other hand, utilizes Dijkstra’s algorithm to compute the service deployment cost, optimizing the deployment strategy by minimizing costs and enhancing the efficiency of edge computing resource utilization. It collaborates with the service allocation module to ensure resources are optimally distributed. Through their seamless integration and collaboration, these two algorithms enable the system to efficiently handle complex computation and communication tasks while maintaining high service quality.

Through the collaborative application of depth-first search and the Dijkstra algorithm, the cost of service deployment among edge servers can be calculated accurately and efficiently, providing important data support for subsequent service dynamic allocation strategies to ensure the rationality and economy of service allocation.

### 4.3. MESDA Service Dynamic Allocation Strategy

Next, this paper will design a service dynamic allocation scheme based on location prediction results and corresponding constraints to optimize resource allocation. In the field of service allocation optimization, the Grey Wolf Optimization algorithm (GWO) has become an effective method due to its simulation of the predatory behavior of grey wolf packs. Among them, the Alpha wolf represents the currently found optimal solution, followed by the Beta wolf and the Delta wolf, and other wolf individuals continuously update their positions under their guidance in an attempt to find the globally optimal service allocation scheme. However, in a mobile edge computing network with a complex geographical environment and diverse user needs, the traditional GWO algorithm gradually exposes serious limitations. When facing a multi-modal state in service allocation problems, the Alpha wolf often falls into a local optimal solution.

To overcome these shortcomings of the GWO algorithm in complex scenarios, we propose an innovative improved strategy, MESDA (Modified Grey Wolf Optimization for Service Dynamic Allocation), a service dynamic allocation strategy. Its core idea is to introduce an adaptive mechanism, by dynamically adjusting the key parameters in the algorithm to enhance the search ability of the algorithm and the ability to jump out of local optima, so as to better adapt to the complex and changeable service allocation needs in the mobile edge computing scenario.

In the MESDA algorithm, the dynamic adjustment of two key parameters—vector A and vector C—plays a central role, and their changes can significantly change the search behavior and direction of the algorithm.

First, we define the current iteration number as *t* and the total iteration number as *T*. Based on these two variables, we calculate the attenuation factor *a* in each iteration process, and the calculation formula is as follows:

(21) $$a=2-\frac{2t}{T}\;$$

The value range of *a* is between [0,2], and it decreases linearly as the iteration progresses. It represents the “momentum” of the search direction in each iteration. The later the iteration number, the larger the momentum value, and the stronger the ability to adjust the direction. This gradual decrease ensures that early iterations focus more on exploration, while later iterations can better refine and exploit the search directions, enhancing convergence. Based on this attenuation factor, we further calculate the value of vector *A*:

(22) $$A=2a\times r_{a}-a$$

Here, $r_{a}$ is a random vector within the range of [0,1], and *a* is the attenuation factor. The vector *A* can continuously change the search direction during the iteration process. In each iteration, the random vector $r_{a}$ will be regenerated, so that the algorithm can explore different regions in different iteration stages, effectively preventing the algorithm from converging to a local optimal solution too early. The random factor ra ensures that the search process remains dynamic and diverse, allowing the algorithm to avoid premature convergence to suboptimal solutions and explore a broader solution space. For the vector *C*, it mainly controls the surrounding behavior of the algorithm near the optimal solution. We introduce a random factor to enhance the randomness of deviation, and the formula is as follows:

(23) $$C=2\times r_{c}$$

Similarly, $r_{c}$ is a random vector within the range of [0,1], and the algorithm can adjust the search range more flexibly near the optimal solution. The random factor *r_c_* introduces flexibility in the search near the optimal solution, allowing for fine-tuning and adjustment, which helps the algorithm escape local optima and refine the search toward the global optimum.

The above has introduced the specific formula improvements for dynamically adjusting vector A and vector C. From the perspective of heuristic algorithm application, dynamically adjusting A and C not only represents the gradual iteration and optimization of the direction vector and distribution vector, but also balances the relationship between exploration and exploitation in the iteration process. The pseudo-code of the improved Grey Wolf Optimization algorithm is shown in Algorithm 3.
**Algorithm 3** Grey Wolf Optimization Algorithm with Adaptive Parameters.  1:For all *i*, initialize the positions $X_{i}$ of the grey wolf population;  2:Initialize *a*, *A* and *C*;  3:Initialize the positions of Alpha, Beta and Delta wolves as the three best solutions;  4:Define the maximum number of iterations *T*;  5:**for** each generation t=1,2,…,T **do**  6: Adaptively update the *a* parameter: $a=2-\frac{2t}{T}$;  7: **for** each wolf *i* **do**  8:  Update A=2a·rand()−a;  9:  Update C=2·rand();10:  Update the position $X_{i}$ of the wolf;11: **end for**12: Evaluate the fitness of the wolf pack and update Alpha, Beta and Delta wolves;13:**end for**14:**return** the best solution

It can be seen from the following pseudo-code that this paper implements the automated search of two search parameters: including the search direction parameter A and the search surrounding parameter C in each iteration step. Compared with the original Grey Wolf Optimization algorithm, in each iteration step, through the update of the new generation of wolves, for each wolf in the algorithm wolf pack, its search parameters are updated once. An additional algorithm with a complexity of O(m×n) is introduced, where *m* represents the number of iterations and *n* represents the number of wolves in the pack, introducing only a small additional computation. In addition, the position of each wolf is also updated once in each iteration. Combined with the iteration process of the above Spatial-Temporal Graph Neural Network, it is not difficult to find that compared with the graph structure introduced by the former, the Grey Wolf Optimization algorithm with adaptive parameters actually completes the parameter adaptation with only a very small part of the calculation amount introduced. And for experiments, this update process is simple and efficient: in the comparative experiment stage, we only need to set an adaptive switch (the on state represents enabling adaptive update, and the off state represents not enabling), and we can conveniently conduct experiments in combination with the structure of the spatio-temporal graph network without changing any input and output to obtain the comparative experiment results.

After obtaining the above experimental parameters *A* and *C*, the next step is to embed these two vectors into GWO so that it can exert the effect of exploration/exploitation. Here, this paper borrows the usage of the parameter τ in the process of constructing the DDPG framework using reinforcement learning: the parameter τ is mainly used to balance the source network and the target network in the Actor-Critic framework, where the source network is used for exploration in the experience pool, and the target network is derived from the source network and is responsible for reusing and deepening the obtained experience during the training process. The above-obtained vectors *A* and *C* can also be embedded into GWO by referring to a similar idea. The embedding steps are as follows:

(1) Before updating the position of each wolf, calculate the values of vectors *A* and *C*;
(2) Perform unified normalization on the values of *A* and *C*, that is,
  (24) $$A=\mathrm{normalized}\left(\frac{A}{A+C}\right)\;$$
  (25) $$C=\mathrm{normalized}\left(\frac{C}{A+C}\right)\;$$
  where the normalized method uses the ‘min-max scaler‘ in the ‘sklearn‘ package, and its function is to normalize the values of *A* and *C* to the range (0,1);
(3) Embed the parameters *A* and *C* when simulating the surrounding behavior of the wolf pack, which is expressed by the following two formulas:
  (26) $$D=C\times X_{p(t)}-X_{t}\;$$
  (27) $$X_{t+1}=X_{p(t)}-A\times D\;$$
  where *t* represents the current iteration number, *A* and *C* are the exploration parameter and the exploitation parameter, respectively, $X_{p}$ is the position of the prey, and $X_{t}$ is the position of the grey wolf individual in the *t*-th generation. After the update, the improved GWO method with dynamic factors *A* and *C* is obtained, denoted as MESDA.

By introducing the dynamic adjustment mechanism, the improved GWO is theoretically guaranteed to have strong convergence. As the number of iterations increases, the improved algorithm is able to converge to a stable solution within a finite time. Specifically, as the parameter A gradually decreases and C continues to dynamically update, the search space of the algorithm gradually narrows, eventually converging to a solution near the optimal. This convergence process is supported by the variations of A and C, ensuring the stability of the algorithm.

Through the above analysis and derivation, this paper has realized the preliminary framework of an improved Grey Wolf Optimization algorithm with dynamic exploration vectors (i.e., MESDA). In this framework, this paper has realized the dynamic adjustment and calculation of two important vectors in the Grey Wolf Optimization algorithm process: Vector A is responsible for directionality during the optimization process, and its main goal is exploration, and it guides the objective of model solving towards a better direction. In the calculation process, a step iteration-related factor is also introduced, so that the change of this vector can have stronger adjustability as the number of iterations changes; Vector C is responsible for equilibrium during the optimization process, and its main goal is utilization, aiming to make more model solving results fall closer to the optimal value, thereby ensuring the average effect of model inference. In the calculation process, this paper directly uses random factors to replace C, aiming to weaken the bias of the fixed variable C on the surrounding effect, thereby reducing the variance of the distribution of calculation results.

## 5. Experiment

This chapter aims to explore, through experiments, the impact of the proposed service dynamic allocation strategy based on location prediction on the optimization objectives in specific mobile edge computing scenarios. First, some settings of the experiments in this paper are introduced, including datasets, experimental parameters, and evaluation indicators, as well as software and hardware environment configurations, etc. Then, the advantages of the ELPM + MESDA method are verified through control experiments from the perspectives of the location prediction model and the service allocation strategy.

### 5.1. Dataset

This study selects the Shanghai Telecom base station dataset [38] as the dataset of edge servers, which covers the accurate locations of 3233 Shanghai Telecom base stations and over 7.2 million Internet access records of 9481 mobile phones within 6 consecutive months, and each record contains the detailed start time and end time of each mobile user’s access to the base station. In this paper, these base stations are considered as edge servers.

The “month” field in the dataset indicates the month when the mobile user records were made, while “date” refers to the specific date of the record. “Start time” and “end time” represent the exact start and end times of the record, respectively. “User ID” refers to the mobile user ID connected to the telecom base station, and “latitude” and “longitude” correspond to the precise geographic coordinates of each base station.

For the edge users, we select the Shanghai taxi trajectory dataset [38], which contains the trajectories of 4328 taxis in Shanghai on 20 February 2007. The dataset records the specific location information of vehicles every 1 to 1.5 min throughout a single day. The data include the vehicle ID, date and time, vehicle longitude, vehicle latitude, vehicle speed (km/h), vehicle heading, and passenger status (0 for no passenger, 1 for with passenger). For the purposes of this study, we select five pieces of information—date and time, vehicle longitude, vehicle latitude, vehicle speed, and vehicle heading—as input parameters for the ELPM (Edge Location Prediction Model).

Additionally, we choose the data from 8:00 a.m. to 8:30 a.m. on the selected day to conduct dynamic service allocation for users in the context of the morning rush hour. The training and testing datasets are split in a 4:1 ratio. The data from 8:00 a.m. to 8:24 a.m. are used as the training set, while the data from 8:25 a.m. to 8:30 a.m. are used as the test set. The goal is to predict the specific geographic coordinates (latitude and longitude) of each edge user during the period of 8:25 a.m. to 8:30 a.m., which will be used in the next stage of the service allocation process.

The experiments simulate various scenarios by varying the number of edge users (15, 30, 45, 60, 75), the number of edge servers (300, 400, 500, 600, 700), and the initial resource capacity of the edge servers (50%, 75%, 100%, 125%, 150%). During the sampling process, the number of data points is counted by vehicle ID and User ID, and users and servers are divided into four equal-sized buckets to ensure comprehensive and objective sampling.

In our experiment, base station locations and taxi trajectory data are primarily employed to simulate users’ mobility patterns. These datasets facilitate the understanding of users’ behavioral characteristics across different times and locations, thereby providing a foundational basis for location prediction and service allocation. Meanwhile, such data indirectly influence server deployment, as the locations and coverage ranges of servers are determined based on users’ mobility paths and density distributions. Thus, the dataset not only impacts the modeling of users’ mobility patterns but also shapes the distribution of edge servers and the formulation of service optimization strategies.

Three evaluation metrics are used: (1) The higher the percentage of users connected to edge servers, the better, reflecting service coverage; (2) the lower the average service deployment cost, the better, which relates to resource utilization; and (3) the shorter the execution time of the service allocation strategy, the better, reflecting efficiency. The first two metrics evaluate effectiveness, while the last one assesses efficiency.

### 5.2. Control Experiment

This paper divides the control experiments into two parts: the location prediction model control group and the service allocation strategy control group. For each group, 4 to 5 sets of control experiments are designed to explore the strengths and weaknesses of the proposed method. Following the principle of controlling variables, the experimental setups for each control group are shown in Table 2.

#### 5.2.1. Control Group of Location Prediction Model

This subsection mainly explores the impact of location prediction models on the entire experiment. The location prediction control experiment groups selected from the experimental groups Set #2.1∼2.3 are as follows:

- ELPM: This refers to the location prediction model proposed in this paper for mobile edge computing environments. For the ELPM model, we used the following hyperparameters: a learning rate of 0.001, a batch size of 32, and 100 hidden layers.
- STNN: This is the Spatial-Temporal Neural Network model, which effectively combines the features of temporal data and spatial data. Using this model for location prediction is suitable for mobile edge computing scenarios and has certain typical advantages. By comparing with STNN, we are able to verify the accuracy and efficiency of our method in predicting user locations in dynamic environments.
- PDFormer [39]: This model utilizes a spatial self-attention module to capture dynamic spatial dependencies, introduces two graph masking matrices to highlight short- and long-range spatial dependencies, and uses a traffic delay-aware feature transformation module to accurately simulate the time delay in spatial information propagation, providing relatively accurate traffic prediction results. Choosing PDFormer as a baseline helps us compare the performance differences between self-attention-based models and our Spatial-Temporal Graph Neural Network approach, especially in modeling complex spatio-temporal dependencies.
- D^2^STGNN [8]: This model is a decoupled dynamic Spatial-Temporal Graph Neural Network that captures spatio-temporal correlations and learns dynamic features of traffic networks through a dynamic graph learning module. Choosing D^2^STGNN as a baseline can demonstrate the advantages of our method in dynamic spatio-temporal modeling and learning dynamic features.
- LSTM: This is the Long Short-Term Memory network model, which effectively captures long-term dependencies and temporal features in sequential data in mobile edge computing scenarios. It can also learn abstract features of data through a multi-layer neural network structure, thereby improving the accuracy of location prediction. Choosing LSTM as the benchmark model can help us compare the effects of traditional time-series prediction methods and our innovative method based on the Spatial-Temporal Graph Neural Network.

**Figure 3:**
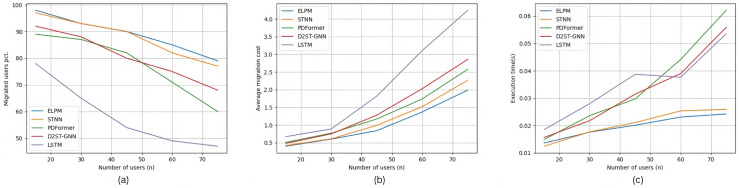
Experimental results of Set #2.1.

**Figure 4:**
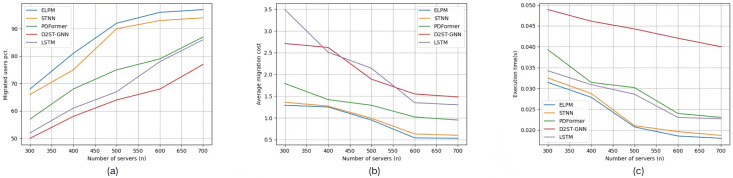
Experimental results of Set #2.2.

**Figure 5:**
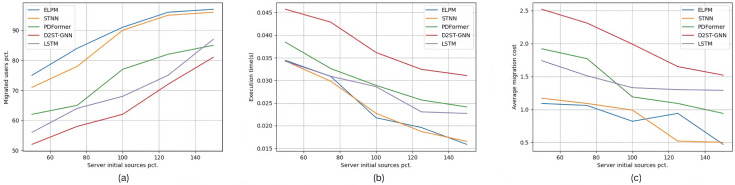
Experimental results of Set #2.3.

The results of experimental groups Set #2.1∼2.3 are shown in Figure 3, Figure 4 and Figure 5. The three images (a), (b), and (c) in Figure 3 show the experimental results of experimental group Set #2.1, where the number of edge users increased from 15 to 75. In the five specific scenarios shown in Figure 3a, the percentage of edge users connected to the edge servers using ELPM is the highest among all experimental control groups, averaging 89%. STNN is very close to this value, with an average of 87.8%. The next in line is D2STGNN, with 80.6%, followed by PDFormer with 77.8%. The worst performer in this scenario is LSTM, with only 58.6%. Accordingly, in Figure 3b, the average service deployment cost per edge user for each location prediction method in the five specific scenarios, in the order from top to bottom as shown in the legend, is 1.04, 1.162, 1.354, 1.48, and 2.148. Moreover, as the number of edge users increases, there is a noticeable trend of an increasing rate of service deployment cost. In Figure 3c, the average execution time of the service dynamic allocation strategy for each location prediction method in the five specific scenarios, in the order from top to bottom as shown in the legend, is 0.01969 s, 0.02047 s, 0.034898 s, 0.032746 s, and 0.035284 s.

The three images (a), (b), and (c) in Figure 4 show the experimental results of experimental group Set #2.2, where the number of edge servers increased from 300 to 700. In Figure 4a, for the five specific scenarios, the percentage of edge users connected to edge servers using each location prediction method, in the order from top to bottom as shown in the legend, is 86.8%, 83.6%, 73.2%, 63.4%, and 68.8%, respectively. In Figure 4b, the average service deployment cost per edge user for each location prediction method in the five specific scenarios is 0.912, 0.97, 1.294, 2.05, and 2.158, respectively. In Figure 4c, the average execution time of the service dynamic allocation strategy for each location prediction method in the five specific scenarios is 0.02334 s, 0.024138 s, 0.029588 s, 0.044274 s, and 0.027902 s, respectively. ELPM also achieved the best results among all the control experiments in this paper.

The three images (a), (b), and (c) in Figure 5 show the experimental results of experimental group Set #2.3, where the initial resource capacity of the edge servers increased from the original 50% to 150%. In Figure 5a, for the five specific scenarios, the percentage of edge users connected to edge servers using each location prediction method, in the order from top to bottom as shown in the legend, is 88.6%, 86%, 74.2%, 65%, and 70%, respectively. In Figure 5b, the average service deployment cost per edge user for each location prediction method in the five specific scenarios is 0.876, 0.854, 1.382, 1.998, and 1.434, respectively. In Figure 5c, the average execution time of the service dynamic allocation strategy for each location prediction method in the five specific scenarios is 0.02451 s, 0.024418 s, 0.029952 s, 0.03765 s, and 0.027902 s, respectively.

The average values of the evaluation metrics obtained from the combination of each location prediction method and service allocation strategy in all specific mobile edge computing scenarios of experimental groups Set #2.1∼2.3 are shown in Table 3.

In our experiments, the execution time of 0.022 s is lower than the 0.030 s of the LSTM model. In scenarios with high user density and frequent task requests, shorter processing times mean that the system can respond to user requests more quickly, reduce latency, and, thereby, improve user experience. Additionally, a shorter execution time helps avoid resource bottlenecks caused by excessive computation, increases the system’s throughput and stability, and ensures that the system can operate smoothly under high load, meeting the demands of real-time computation and service allocation. Therefore, further optimizing the processing time will make the system more adaptable, enabling it to support larger-scale user and task requests.

Overall, the combination of ELPM + MESDA ranks first in all evaluation metrics among the control methods, demonstrating the excellent adaptability of the proposed method in this scenario and highlighting the necessity of the improvements. In this context, ELPM outperforms other location prediction methods, proving its superior ability to accurately predict user locations compared to alternatives. The combination of 65 follows closely, with evaluation metrics very similar to those of ELPM + MESDA, also showing strong competitiveness in this scenario. When considering only the location prediction methods, PDFormer outperforms D2STGNN, while LSTM has a shorter service allocation execution time compared to PDFormer and D2STGNN. However, LSTM still shows some gaps in other evaluation metrics.

#### 5.2.2. Control Group of Service Allocation Strategy

This subsection mainly explores the impact of service allocation strategies on the entire experiment. The service allocation control experiment groups selected from the experimental groups Set #3.1∼3.3 are as follows:

- MESDA: This refers to the service allocation strategy proposed in this paper for mobile edge computing environments. We set the following parameters for MESDA: a population size of 50, providing a balance between exploration and computational cost, and a maximum of 200 iterations, allowing sufficient time for the optimization process to converge.
- GWO: This is the Grey Wolf Optimizer, which is characterized by fast convergence and strong global search ability. It is suitable for optimizing and adjusting complex problems and can be effectively applied to the dynamic service allocation problem in mobile edge computing environments.
- MDQP [40]: This method considers the service request time and location of mobile clients, uses multi-dimensional indicators to predict service quality, and, thereby, achieves effective service recommendation for mobile clients. This method is referred to as MDQP in this paper.
- K Nearest: This is the k-Nearest Neighbors algorithm, which can select the K nearest neighbor nodes for service resource allocation based on demand in mobile edge computing scenarios.
- Logistic Regression: This is the Logistic Regression model, suitable for solving service allocation problems in mobile edge computing environments.
- Random Forest: This is the Random Forest algorithm, which can improve the accuracy and robustness of service allocation in this scenario by using the ensemble prediction ability of multiple decision trees.

**Figure 6:**
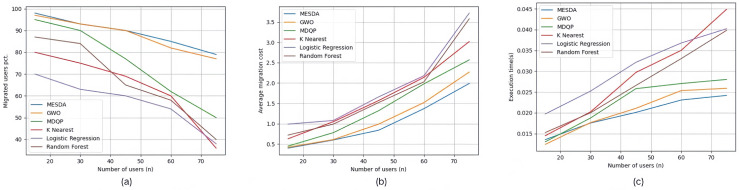
Experimental results of Set #3.1.

**Figure 7:**
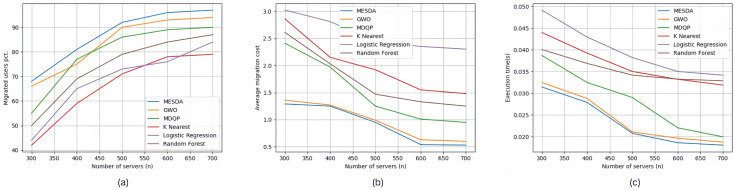
Experimental results of Set #3.2.

**Figure 8:**
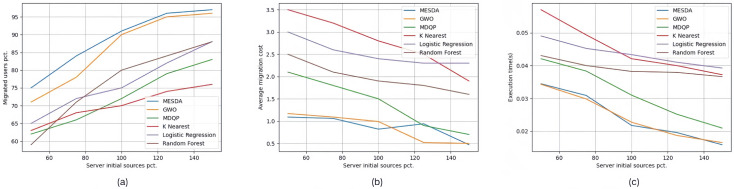
Experimental results of Set #3.3.

The results of experimental groups Set #3.1∼3.3 are shown in Figure 6, Figure 7 and Figure 8.

The three images (a), (b), and (c) in Figure 6 show the experimental results of experimental group Set #3.1, where the number of edge users increased from 15 to 75. In Figure 6a, for the five specific scenarios, the percentage of edge users connected to edge servers using each service allocation strategy, in the order from top to bottom as shown in the legend, is 89%, 87.8%, 74.8%, 64%, 57%, and 66.8%, respectively. In Figure 6b, the average service deployment cost per edge user for each service allocation strategy in the five specific scenarios is 1.04, 1.162, 1.42, 1.682, 1.926, and 1.768, respectively. In Figure 6c, the average execution time for each service allocation strategy in the five specific scenarios is 0.01969 s, 0.02047 s, 0.022526 s, 0.028864 s, 0.030814 s, and 0.02694 s, respectively.

The three images (a), (b), and (c) in Figure 7 show the experimental results of experimental group Set #3.2, where the number of edge servers increased from 300 to 700. In Figure 7a, for the five specific scenarios, the percentage of edge users connected to edge servers using each service allocation strategy, in the order from top to bottom as shown in the legend, is 86.8%, 83.6%, 79.4%, 65.8%, 68.4%, and 73.8%, respectively. In Figure 7b, the average service deployment cost per edge user for each service allocation strategy in the five specific scenarios is 0.912, 0.97, 1.518, 1.992, 2.584, and 1.738, respectively. In Figure 7c, the average execution time for each service allocation strategy in the five specific scenarios is 0.02334 s, 0.024138 s, 0.028438 s, 0.036674 s, 0.039908 s, and 0.03545 s, respectively.

The three images (a), (b), and (c) in Figure 8 show the experimental results of experimental group Set #3.3, where the initial resource capacity of the edge servers increased from the original 50% to 150%. In Figure 8a, for the five specific scenarios, the percentage of edge users connected to edge servers using each service allocation strategy, in the order from top to bottom as shown in the legend, is 88.6%, 86%, 72.4%, 70.2%, 76.4%, and 76.4%, respectively. In Figure 8b, the average service deployment cost per edge user for each service allocation strategy in the five specific scenarios is 0.876, 0.854, 1.4, 2.78, 2.52, and 1.98, respectively. In Figure 8c, the average execution time for each service allocation strategy in the five specific scenarios is 0.02451 s, 0.024418 s, 0.031498 s, 0.045124 s, 0.043564 s, and 0.03916 s, respectively.

The average values of the evaluation metrics obtained from the combination of each location prediction model and service allocation strategy in all specific mobile edge computing scenarios of experimental groups Set #3.1∼3.3 are shown in Table 4.

Based on the above experimental results, it is evident that MESDA outperforms other service allocation strategies in terms of a higher percentage of edge users connected to edge servers, lower service deployment cost, and shorter service allocation execution time. The reasons why its execution time is shorter than that of K Nearest, Logistic Regression, and Random Forest are as follows: Firstly, both ELPM and MESDA can perform inference within a single epoch, while K Nearest, Logistic Regression, and Random Forest are machine learning methods that require multiple iterations of data feeding and adjustment to ensure experimental performance, thus requiring multiple epochs for inference. Secondly, ELPM and MESDA are implemented based on PyTorch (version 1.10), which provides faster inference compared to the sklearn implementations of the other three methods.

Overall, the combination of ELPM and MESDA (the improved combination) has seen a 2.56%, 5.29%, and 2.16% improvement in the three evaluation metrics, respectively, compared to the combination of STNN and GWO (the original combination). The MESDA strategy, in particular, shows its effectiveness in optimizing service allocation, contributing to the overall performance improvement. The experimental results prove that the method proposed in this paper performs better in this scenario and demonstrate the feasibility and superiority of the improved approach.

#### 5.2.3. Limitations and Future Work

**Figure 9:**
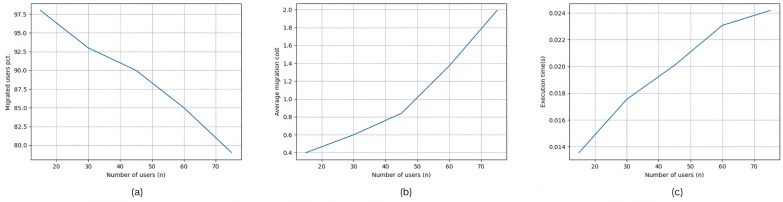
Experimental results of Set #2.1 with varying number of edge users. (**a**) Edge user connection rate drops from 98% to 79%. (**b**) Average deployment cost per user rises from 0.4 to 1.99. (**c**) Execution time increases from 0.01356 s to 0.02418 s due to rising demand.

Figure 9 shows the experimental results of Set #2.1, where the number of edge users increased from 15 to 75. In Figure 9a, the percentage of edge users connected to edge servers decreases from 98% to 79% as user numbers rise. In Figure 9b, the average service deployment cost per edge user increases from 0.4 to 1.99, and in Figure 9c, the execution time for dynamic service allocation rises from 0.01356 s to 0.02418 s. These changes occur because the limited resources of edge servers cannot keep up with increasing demand, leading to users being disconnected, higher service deployment costs, and longer execution times.

While the ELPM model performs well in location prediction and service allocation, practical deployment faces challenges. The computational overhead on edge devices could cause delays and energy issues, especially when processing large real-time datasets. Additionally, as user numbers increase, scalability becomes a concern, particularly in dense networks (e.g., 10,000+ users) [41]. Furthermore, in low-mobility scenarios, where users do not exhibit significant movement, the model’s performance may degrade. This is because the lack of substantial movement reduces the effectiveness of location prediction, which, in turn, can negatively impact the service allocation efficiency. In such scenarios, the advantage of predictive modeling diminishes, making the system less responsive to user demands. Future work will focus on optimizing efficiency, exploring lightweight versions of ELPM, and using distributed computing and load balancing strategies to improve scalability and system stability.

## 6. Conclusions

This article proposes a location prediction-based dynamic service allocation strategy (MESDA) to address the issue of dynamic service allocation in mobile edge computing (MEC) environments. The strategy formulates the problem as a dynamic optimization problem, aiming to minimize service deployment costs while maximizing the percentage of edge users connected to edge servers, taking into account user mobility and the dynamic nature of service demands. Experimental results show that in scenarios with high user mobility and varying service demands, the proposed method outperforms static models in terms of connection rates and service allocation efficiency. However, in environments with low user mobility and stability, static allocation strategies outperform the dynamic model in execution time due to their simpler computation. 

## Figures and Tables

**Table 1:** Symbols for the dynamic allocation problem of edge user services.

Notation	Description
$a_{i}$	$a_{i}=\langle a_{i}^{1},a_{i}^{2},\ldots ,a_{i}^{d}\rangle$, the remaining available resources of edge server $s_{i}$, where $a_{i}^{q}$ represents one of the resources (CPU, storage, bandwidth, etc.), q∈D
*D*	D={CPU, storage, bandwidth}, the available resource types of edge servers
$cov_{i}$	The geographic area radius that edge server $s_{i}$ can cover, within which edge users may connect to this server to obtain services
$dc_{u}$	The service deployment cost generated by edge user *u* migrating service $sc_{u}$
$h_{i,j}$	The number of hops on the shortest network path between edge servers $s_{i}$ and $s_{j}$, used to measure the transmission distance between servers
$h_{u}$	The delay experienced by edge user *u* when connected to a remote cloud
$mc_{u}$	The total cost integrating the migration cost and delay cost of edge user *u*
*R*	$R=\{r_{1},r_{2},\ldots ,r_{v}\}$, a set of predefined resource consumption amounts of service $sc_{p}$, where $r_{p}=\langle r_{p}^{1},r_{p}^{2},\ldots ,r_{p}^{d}\rangle$, and $r_{p}^{q}$ represents the consumption amount of one of the resources, q∈D
*S*	$S=\{s_{1},s_{2},\ldots ,s_{m}\}$, a finite set composed of edge servers $s_{i}$
*U*	$U=\{u_{1},u_{2},\ldots ,u_{n}\}$, a finite set of edge users whose required services have the possibility of being allocated
SC	$SC=\{sc_{1},sc_{2},\ldots ,sc_{v}\}$, a finite set composed of services $sc_{p}$
S(u)	A finite set of edge servers whose service range covers edge user *u*
$sc_{u}$	A service required by edge user *u*, $sc_{u}\in SC$
$S(sc_{u})$	A finite set of edge servers that own the service $sc_{u}$ required by edge user *u*
$\tilde{U}\left(s_{i}\right)$	A finite set of edge users whose required services are successfully allocated to edge server $s_{i}$
$x_{u,i}$	Whether the required service of edge user *u* is allocated to edge server $s_{i}$ ($x_{u,i}=1$) or not ($x_{u,i}=0$)
$y_{i,p}$	Whether edge server $s_{i}$ owns service $sc_{p}$ ($y_{i,p}=1$) or not ($y_{i,p}=0$)

**Table 2:** Experimental setup (controlled experiment).

	Edge Users	Edge Servers	Initial Resources of Edge Servers	Location Prediction Model + Service Allocation Strategy
				ELPM + MESDA
Set #2.1	15, 30, 45, 60, 75	500	100%	STNN + GWO
Set #2.2	45	300, 400, 500, 600, 700	100%	PDFormer + MESDA
Set #2.3	45	500	50%, 75%, 100%, 125%, 150%	D^2^STGNN + MESDA
				LSTM + MESDA
				ELPM + MESDA
Set #3.1	15, 30, 45, 60, 75	500	100%	STNN + GWO
Set #3.2	45	300, 400, 500, 600, 700	100%	ELPM + MDQP
Set #3.3	45	500	50%, 75%, 100%, 125%, 150%	ELPM + K Nearest
				ELPM + Random Forest

**Table 3:** Experimental setup (controlled experiment).

	Average Percentage of Users Connected to Edge Servers	Average Service Setup Cost	Average Service Allocation Execution Time
ELPM + MESDA	88%	0.942667	0.022513 s
STNN + GWO	85.80%	0.995333	0.023009 s
PDFformer + MESDA	75.07%	1.343333	0.031479 s
D^2^STGNN + MESDA	69.67%	1.842667	0.038223 s
LSTM + MESDA	65.80%	1.913333	0.030363 s

**Table 4:** Experimental setup (controlled experiment).

	Average Percentage of Users Connected to Edge Servers	Average Service Setup Cost	Average Service Allocation Execution Time
ELPM + MESDA	88%	0.942667	0.022513 s
STNN + GWO	85.80%	0.995333	0.023009 s
ELPM + MDQP	75.53%	1.446	0.027487 s
ELPM + K Nearest	67%	2.151333	0.036887 s
ELPM + Logistic Regression	67%	2.343333	0.038095 s
ELPM + Random Forest	72.33%	1.828667	0.033855 s

## Data Availability

The original contributions presented in this study are included in the article. Further inquiries can be directed to the corresponding author.
